# A new species of nectar-feeding bat, genus *Lonchophylla*, from the Caatinga of Brazil (Chiroptera, Phyllostomidae)

**DOI:** 10.3897/zookeys.514.10013

**Published:** 2015-07-22

**Authors:** Ricardo Moratelli, Daniela Dias

**Affiliations:** 1 Fiocruz Mata Atlântica, Fundação Oswaldo Cruz, CEP 22713-375, Rio de Janeiro, Brazil; 2 Division of Mammals, National Museum of Natural History, Smithsonian Institution, P.O. Box 37012, Washington, DC 20013-7012, U.S.A.; 3 Laboratório de Biologia e Parasitologia de Mamíferos Silvestres Reservatórios, Instituto Oswaldo Cruz, CEP 21040-900, Fundação Oswaldo Cruz, Rio de Janeiro, Brazil

**Keywords:** Atlantic Forest, Caatinga, Cerrado, *Lonchophylla inexpectata*, *Lonchophylla dekeyseri*, *Lonchophylla mordax*, North-eastern Brazil

## Abstract

We describe *Lonchophylla inexpectata* **sp. n.** from the Caatinga of Brazil. This new species can be distinguished from all known species of *Lonchophylla* that occur in Brazil by dental traits, cranial size, and fur colour. Specimens of *Lonchophylla inexpectata* have been misidentified as *Lonchophylla mordax*; but *Lonchophylla inexpectata* is a pale-venter species, similar in external appearance to *Lonchophylla dekeyseri*. We have found *Lonchophylla inexpectata* in the Caatinga of North-eastern Brazil; *Lonchophylla mordax* along the eastern border of the Caatinga and in the Atlantic Forest–Caatinga ecotone in North-eastern Brazil; and *Lonchophylla dekeyseri* in the Cerrado of Mid-western Brazil, in the Brazilian Cerrado–Caatinga ecotone, and as far west as the Cerrado of Bolivia.

## Introduction

*Lonchophylla* Thomas, 1903 (Phyllostomidae) comprises 12 species of nectar-feeding bats restricted to the Neotropics (Griffiths and Gardner 2008, Parlos et al. 2014). Parlos et al. (2014) revised the Lonchophyllinae and established *Hsunycteris* as a new genus to include the smaller species formerly known as the *Lonchophylla thomasi* complex. However, their revision did not include the Brazilian species *Lonchophylla mordax* Thomas, 1903, *Lonchophylla bokermanni* Sazima et al., 1978, *Lonchophylla dekeyseri* Taddei et al., 1983, and *Lonchophylla peracchii* Dias et al., 2013. During our assessment of these Brazilian species we found evidence of another new taxon based on specimens from the Brazilian Caatinga we found in museum collections. Some specimens of this previously undescribed species have been misidentified as *Lonchophylla mordax* for more than a century.

*Lonchophylla mordax* was described from Lamarão, Bahia (Thomas 1903), with subsequent records ascribed to specimens from other localities in Northern ([N] Handley 1967, Piccinini 1974, Koopman 1981), North-eastern ([NE] Vieira 1955, Sazima et al. 1978, 1983, Mares et al. 1981, Willig 1983, Astúa and Guerra 2008), Mid-western ([MW] Peracchi et al. 2011), and South-eastern Brazil ([SE] Pereira-Barreto et al. 1968, Taddei et al. 1978, Pedro and Passos 1995, Esbérard et al. 2006, Dias et al. 2002, Esbérard 2003). Handley (1966) synonymized *Lonchophylla concava* Goldman, 1914 under *Lonchophylla mordax*, thus enlarging its geographic distribution westward into western Colombia and Ecuador, and northwestward into Costa Rica. This arrangement was rejected by Albuja and Gardner (2005), who recognized *Lonchophylla concava* as a distinct species. Based on the records available, bat biologists have assumed that *Lonchophylla mordax* was restricted to eastern South America, with records from the Amazon Forest of N Brazil, eastward to xeric habitats in NE Brazil, and southward to the Atlantic Forest of SE Brazil, including transitional areas between these last two biomes (see Griffiths and Gardner 2008, Peracchi et al. 2011).

Thomas’s (1903) description of *Lonchophylla mordax* is based on eight specimens from Lamarão, Bahia collected by Alphonse Robert in 1903. Lamarão is in the *agreste* sub region of NE Brazil, which is a narrow transition zone between the coastal Atlantic Forest to the east and the semiarid Caatinga on the west (Prado 2003). According to local residents, the vegetation in Lamarão and adjacent areas during the first half of the 20th century was dominated by tall forests, which is characteristic of the transitional vegetation between the Atlantic Forest and Caatinga. Throughout the last century, land-use practices have converted the region into a semi-arid environment that resembles caatinga habitats. The type material of *Lonchophylla mordax*, originally deposited in the Natural History Museum, London (BM), includes the holotype (BM 1903.9.5.34) and seven paratypes. One of the paratypes was sent to the Smithsonian’s National Museum of Natural History, Washington, DC (USNM 123392). A few years after Thomas described *Lonchophylla mordax*, a series of *Lonchophylla* were collected in Barra, Bahia by Ernest Garbe and Robert H. Becker in 1908 and 1914, respectively. Barra, Bahia is in the *sertão* sub region (450 to 500 km west of Lamarão), a semi-arid environment that is characteristic of the Caatinga (Prado 2003). According to their labels, Garbe’s and Becker’s specimens from Barra were identified as *Lonchophylla mordax* and either originally deposited or subsequently sent to museums in Brazil and United States of America. This material has been the basis for several subsequent published accounts on *Lonchophylla mordax* (e.g., Lima 1926: 36, Vieira 1942: 321). As with the paratype of *Lonchophylla mordax* (USNM 123392), one of those specimens collected by Garbe is housed in the Smithsonian’s National Museum of Natural History (USNM 238008). After comparing skins and skulls of Garbe’s and Thomas’s USNM specimens from Barra (Caatinga, USNM 238008) and Lamarão (Atlantic Forest/Caatinga, USNM 123392), we determined that the pale-venter *Lonchophylla* from Barra could be distinguished from *Lonchophylla mordax*, and represented an undescribed species. Among distinctive traits distinguishing the Barra specimen from *Lonchophylla mordax* are the paler colour of the ventral fur and the smaller skull that has a narrower and more delicate rostrum.

To test this hypothesis and further understand the geographic distribution of Brazilian species, we examined series of *Lonchophylla* from localities in the Caatinga, Cerrado, and Atlantic Forest, as well as from transitional zones between these habitats. The material used in our comparisons represents all *Lonchophylla* species known to occur in Brazil. During this process we found additional features that support our hypothesis that the pale-venter *Lonchophylla* from the Caatinga represents a new species, which we describe below.

## Methods

The material we used in the comparisons includes series of *Lonchophylla* from the Caatinga of NE Brazil (Bahia [municipalities of Andaraí, Barra, Buíque], Ceará, Pernambuco, Piauí, Sergipe [Grota do Angico]); Cerrado of Bolivia (Santa Cruz) and Mid-western Brazil (Distrito Federal, Goiás, Mato Grosso do Sul); Atlantic Forest of SE Brazil (Espírito Santo, Rio de Janeiro); and the Atlantic Forest–Caatinga ecotone in NE Brazil (Bahia [Lamarão], Sergipe [Itabaiana]). This material includes representatives of all currently recognized Brazilian species of *Lonchophylla*, and includes primary and secondary types of *Lonchophylla bokermanni* (6 specimens from the type series), *Lonchophylla dekeyseri* (holotype and one paratype), *Lonchophylla mordax* (holotype and one paratype), and *Lonchophylla peracchii* (holotype and two paratypes). Vouchers are preserved in the American Museum of Natural History (AMNH, New York, USA); Carnegie Museum of Natural History (CM, Pittsburgh, USA); Museu Nacional (MN, Rio de Janeiro, Brazil); Muséum d’histoire naturelle (MHNG, Geneva, Switzerland); Natural History Museum (BM, London, England); Smithsonian’s National Museum of Natural History (USNM, Washington DC, USA); Universidade Estadual Paulista Júlio de Mesquita Filho (DZSJRP, São José do Rio Preto, Brazil); Universidade Federal do Espírito Santo (UFES, Espírito Santo, Brazil); Universidade Federal Rural do Rio de Janeiro (ALP, LMD, Seropédica, Brazil). A complete list of specimens examined is in the Appendix. Most geographical coordinates follow Gardner’s (2008) gazetteer of marginal localities.

Measurements in this report are from adults, and are either in millimetres (mm) or grams ([g] body mass). The body mass was recorded from skin labels. Other dimensions include: the forearm length (FA), from the elbow to the distal end of the forearm including carpals, measured with the wing partially folded; greatest length of skull (GLS), from the posteriormost point of the occiput to the tips of the upper inner incisors; condylo-incisive length (CIL), from the line connecting the occipital condyles to the tips of the upper inner incisors; basal length (BAL), from the anterior margin of the foramen magnum to the tips of the upper inner incisors; maxillary toothrow length (MTL), from the anterior surface of the upper canine, including the cingulum, to the posterior surface of M3; molariform toothrow length (M1M3), from the crown of M1 to the crown of M3; breadth across canines (BAC), greatest breadth across outer surface of the crowns of upper canines, including cingulae; breadth across molars (BAM), greatest breadth across outer edges of the crowns of upper molars; postorbital breadth (POB), least breadth across frontals posterior to the postorbital bulges; braincase breadth (BCB), greatest breadth of the globular part of the braincase; mastoid breadth (MAB), greatest breadth across the mastoid region; mandibular length (MAL), from the mandibular symphysis to the condyloid process; and the mandibular toothrow length (MAN), from the anterior crown of the lower canine, including cingulum, to the posterior crown of m3. Craniodental measurements were taken under binocular dissection microscopes with low magnification (usually 6×). Dimensions were taken by only one of us, using digital callipers accurate to 0.02 mm. Measurements were recorded and analysed to the nearest 0.01 mm, but values were rounded off to 0.1 mm throughout the text because this is the smallest unit that allows accurate repeatability with callipers (Voss et al. 2013). Descriptive statistics (mean and range) were calculated for all dimensions. The statistical significance of differences among samples was assessed by single analyses of variance (one-way ANOVA). This statistics was performed in PAST (Hamer et al. 2001).

Discriminant Function Analysis (DFA) was used to compare taxa. For the analysis, we selected a subset of the cranial dimensions (GLS, CIL, MAB, BCB, POB, BAC, BAM, M1M3, MTL, MAL) to represent different axes of length and width of the skull. As multivariate procedures require complete datasets, missing values (< 3% of the total dataset) were substituted by means. Measurements were transformed to natural logarithms and the covariance matrices were computed considering all variables. DFA was performed in SPSS.

Nomenclature of tooth morphology follows Phillips (1971). Capitalized colour nomenclature follows Ridgway (1912).

## Taxonomy

### Lonchophylla inexpectata sp. n.

Taxon classificationAnimaliaChiropteraPhyllostomidae

http://zoobank.org/610DFBAE-1726-4666-9B3F-BDCC063D25D2

**Figure 1.**
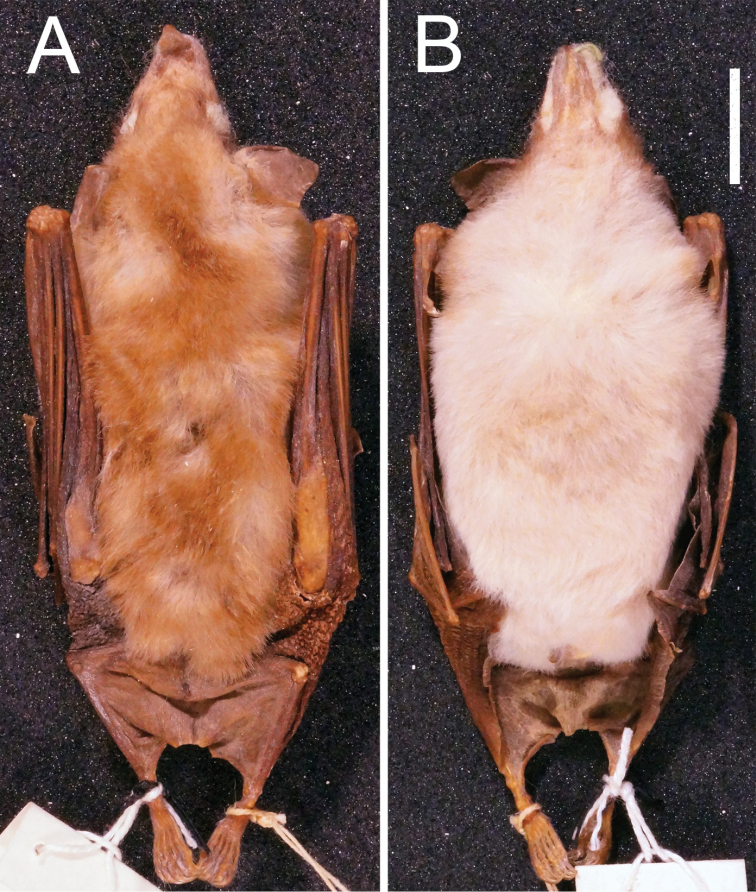
Dorsal **A** and ventral **B** pelage of the holotype of *Lonchophylla inexpectata* (USNM 238008). Scale bar: 10 mm.

**Figure 2.**
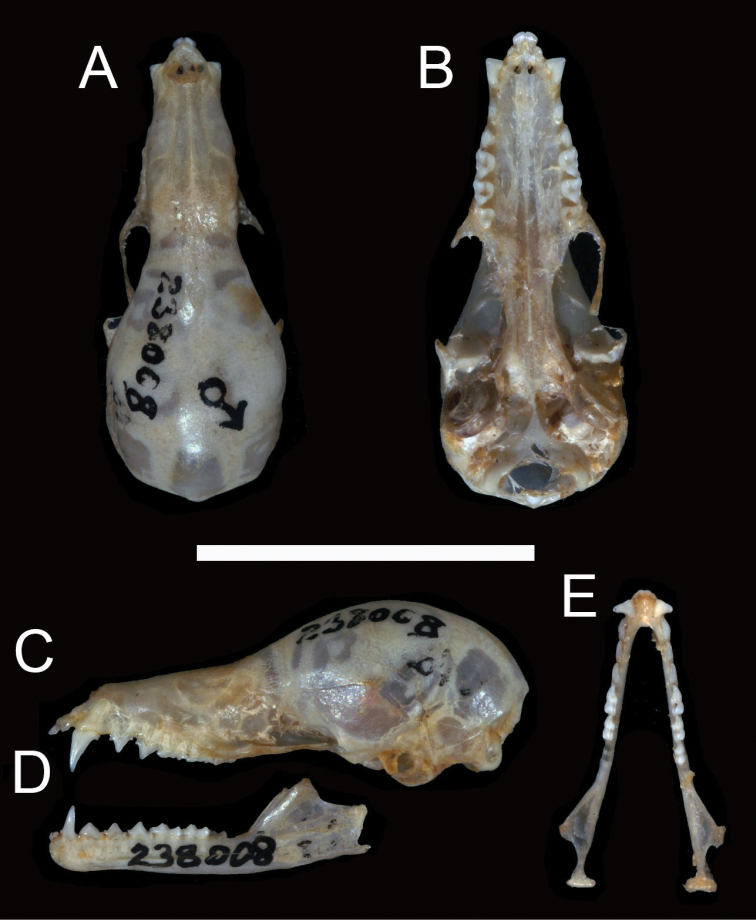
Dorsal **A**, ventral **B**, and lateral **C** views of the cranium, and lateral **D** and dorsal **E** views of the mandible of the holotype of *Lonchophylla inexpectata* (USNM 238008). Scale bar: 15 mm.

Figures 1 , 2 , 4 , 5 ; Table 1

Lonchophylla mordax : Lima 1926: 76; not *Lonchophylla mordax* Thomas, 1903.
Lonchophylla mordax : Vieira 1942: 321; not *Lonchophylla mordax* Thomas, 1903.
Lonchophylla mordax : Taddei, Vizotto and Sazima 1983; not *Lonchophylla mordax* Thomas, 1903.
Lonchophylla dekeyseri : Woodman and Timm 2006: 450; part, not *Lonchophylla dekeyseri* Taddei, Vizotto & Sazima, 1983.
Lonchophylla mordax : Woodman and Timm 2006: 475; part, not *Lonchophylla mordax* Thomas, 1903.
Lonchophylla dekeyseri : Woodman 2007. Part, not *Lonchophylla dekeyseri* Taddei, Vizotto & Sazima, 1983.

#### Holotype.

An adult male, USNM 238008, with skin and skull (Figures 1, 2), including mandible, collected by E. Garbe at Barra (12°42'S, 41°33'W), Bahia, Brazil, on January 1908. Skull and mandible are in good condition except for the minimally damaged anteriormost portion of the foramen magnum. The body is prepared as dry skin. Woodman and Timm (2006: 450) described USNM 238008 as a faded skin, but after comparison of its pelage colour with those from other specimens, only membranes seem to be faded. External and craniodental measurements for the holotype and paratypes are in Table 1.

**Table 1.** Body mass (g) and external and skull measurements (mm) of the holotype (USNM 238008) of *Lonchophylla inexpectata*, and descriptive statistics for *Lonchophylla inexpectata* (from Caatinga [type series]), *Lonchophylla dekeyseri* (from Cerrado), and *Lonchophylla mordax* (from Caatinga and Caatinga–Atlantic Forest ecotone).

	*Lonchophylla inexpectata*	*Lonchophylla inexpectata*	*Lonchophylla dekeyseri*	*Lonchophylla mordax*
	Holotype	Mean	Mean	Mean
	USNM 238008	(Min.–Max.) *N*	(Min.–Max.) *N*	(Min.–Max.) *N*
Body mass	–	**8.2**	–	–
		(7.0–9.5) *15*		
FA	**33.7**	**34.6**	**36.9**	**35.8**
		(32.3–36.4) *62*	(35.5–38.0) *15*	(34.5–37.4) *32*
GLS	**22.3**	**23.1**	22.4***	23.6**
		(22.0–23.9) *38*	(22.0–22.7) *16*	(22.6–24.5) *24*
CIL	**20.8**	**21.7**	21.0***	22.2***
		(20.5–22.6) *37*	(20.4–21.4) *16*	(21.3–23.2) *24*
BAL	**19.1**	**19.8**	19.1***	20.2**
		(18.7–20.7) *36*	(18.5–19.6) *16*	(19.6–20.8) *20*
MTL	**7.6**	**7.8**	7.6**	8.0***
		(7.4–8.2) *45*	(7.3–7.9) *16*	(7.6–8.4) *26*
M1M3	–	**3.3**	3.4*	3.5***
		(3.1–3.6) *40*	(3.3–3.6) *14*	(3.3–3.7) *30*
BAC	**3.4**	**3.6**	3.7**	3.7*
		(3.3–3.8) *44*	(3.4–3.9) *16*	(3.5–4.1) *27*
BAM	**4.8**	**5.1**	**5.1**	5.3*
		(4.8–5.5) *43*	(4.9–5.3) *16*	(4.7–5.7) *26*
POB	**4.1**	**4.3**	4.5***	**4.3**
		(4.1–4.7) *46*	(4.2–4.6) *16*	(4.0–4.6) *27*
BCB	**7.9**	**8.3**	8.4*	**8.5**
		(7.9–8.6) *46*	(8.0–8.7) *16*	(8.1–8.9) *27*
MAB	**8.5**	**9.0**	9.1***	9.3*
		(8.5–9.6) *44*	(8.8–9.4) *16*	(8.9–9.7) *27*
MAL	**14.9**	**15.6**	15.1***	16.1***
		(14.1–16.3) *44*	(14.8–15.4) *16*	(15.5–17.0) *25*
MAN	**8.0**	**8.2**	8.1*	8.4***
		(7.8–8.5) *43*	(7.7–8.4) *16*	(7.9–8.9) *25*

*N* = sample size (adults only, males and females combined). See "Methods" for variable abbreviations and Appendix for localities of specimens used in comparisons. One-way ANOVA for skull measurements is comparing *Lonchophylla inexpectata* with *Lonchophylla dekeyseri* and *Lonchophylla mordax*: * *p* ≤ 0.05, ** *p* ≤ 0.01, *** *p* ≤ 0.001.

#### Paratypes.

The paratype series comprises 46 vouchers. Three paratypes are from the type locality in Barra, Bahia (AMNH 235608, FMNH 21077, 21078), and were collected by R. H. Becker in 1914. One is from Serra do Catimbau, Buíque, Pernambuco (FMNH 137414; 08°37'S, 37°09'W [coordinates for Catimbau National Park]), and was collected by D. Guerra in 1970. Thirty-eight vouchers are from 17 km south of Exu, Pernambuco (CM 99413–99450; 07°41'S, 39°32'W), elevation ca. 480 m, and were collected by M. R. Willig in 1976. Paratypes from Barra (AMNH 235608, FMNH 21077, 21078), and Buíque (FMNH 137414) are in spirits, others are prepared as dry skin.

#### Other specimen.

One additional specimen (ALP 3686) from the Caatinga of Andaraí, Bahia may represent *Lonchophylla inexpectata*. The specimen is preserved in spirit, and the dentition is partially worn, preventing its unambiguous identification.

#### Distribution.

**Figure 3.**
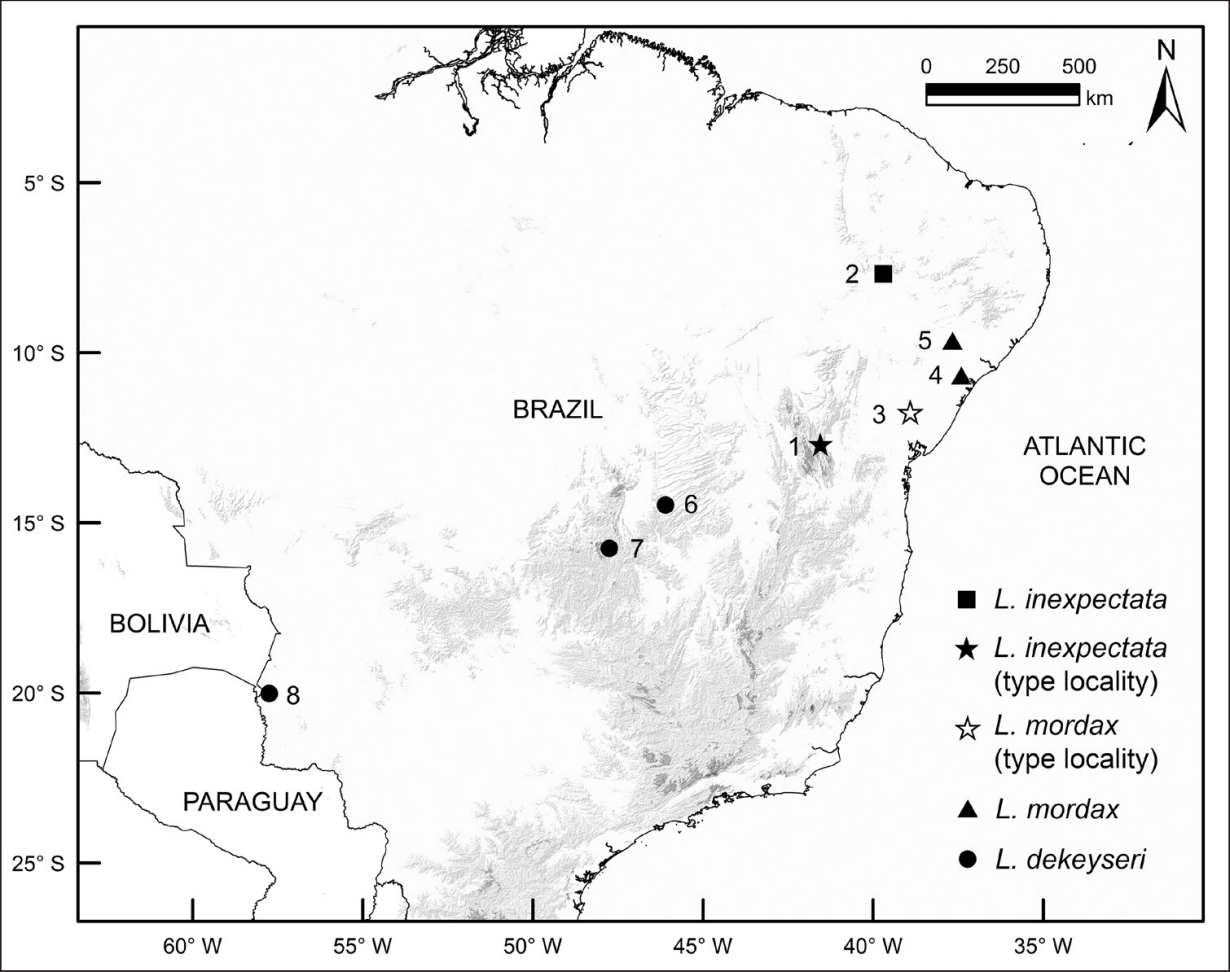
Map of part of South America showing the geographic distribution of samples we confirmed as *Lonchophylla inexpectata* (black star [type locality] and square), *Lonchophylla dekeyseri* (circles), and *Lonchophylla mordax* (white star [type locality] and triangles). Localities 1, 2, 5 are in the Caatinga; localities 3, 4 are in the Caatinga–Atlantic Forest ecotone; and localities 6–8 are in the Cerrado.

*Lonchophylla inexpectata* occurs in the Caatinga of North-eastern (NE) Brazil, with confirmed records from Pernambuco (NE), and Bahia (NE) (Figure 3).

#### Diagnosis.

*Lonchophylla inexpectata* can be distinguished from all South American species that occur east of the Andes by the following set of traits: presence of a lingual cusp in the P4, absence of a lingual cusp in the P3, absence of a deep longitudinal groove in the posterior face of the upper canine, proximal portion of the dorsal surface of the forearm not furred, and ventral fur pale.

#### Description and comparisons.

Like other *Lonchophylla*, the dental formula of *Lonchophylla inexpectata* is 2/2, 1/1, 2/3, 3/3 = 34. *Lonchophylla inexpectata*, *Lonchophylla dekeyseri* and *Lonchophylla bokermanni* are the three pale-venter Brazilian species of the genus, whereas *Lonchophylla mordax* and *Lonchophylla peracchii* have pale-brown ventral pelage. We did not find evidence of *Lonchophylla bokermanni* and *Lonchophylla peracchii* in sympatry with *Lonchophylla inexpectata*—*Lonchophylla bokermanni* is restricted to a small area in the Serra do Espinhaço, Cerrado of Minas Gerais; and *Lonchophylla peracchii* occurs in the Atlantic Forest, from Espírito Santo southward to São Paulo. *Lonchophylla inexpectata* can be distinguished from these two species by the presence of a well-developed lingual cusp in the P4, with lingual root in the median portion of the tooth; absence of a groove along the anterior surface of the upper canines; and proximal portion of the dorsal surface of the forearm not covered with fur.

Based on the samples we have available, *Lonchophylla inexpectata* resembles *Lonchophylla dekeyseri* in the pale ventral fur, and *Lonchophylla mordax* in the dental morphology. These three species overlap partially in external and cranial size, but in general, cranial measurements for *Lonchophylla inexpectata* average significantly larger than those for *Lonchophylla dekeyseri* and smaller than those for *Lonchophylla mordax* (Table 1).

*Lonchophylla mordax* has been reported in the literature as a pale-venter species (e.g., Lima 1926, Vieira 1942, Taddei et al. 1983, Nogueira et al. 2007), and subsequent to the description of *Lonchophylla dekeyseri*, these taxa have been considered the two pale-venter species from NE Brazil (see Taddei et al. 1983, Nogueira et al. 2007, Dias et al. 2013). However, after examining part of the type series of *Lonchophylla mordax* (BM 1903.9.5.34 [holotype], USNM 123392 [paratype]), along with one other specimen from the same locality of the type series (MHNG 667.13 [identified as *Lonchophylla mordax* by Thomas]), and samples from a nearby locality having similar habitat (Itabaiana, Sergipe)—whose external and skull morphology fit with those of the type series of *Lonchophylla mordax* (ALP 8768–8770, 8812–8819)—we concluded that *Lonchophylla mordax* has a light-brown ventral pelage, which is consistently darker than the paler ventral pelage of the type material of *Lonchophylla dekeyseri* and other samples of this species. The ventral pelage of specimens from Barra, Bahia (*Lonchophylla inexpectata*) is similar to that of *Lonchophylla dekeyseri*. Under “historical remarks” we discuss the reasons for previous assignments of pale-venter samples from the Caatinga of NE Brazil (= *Lonchophylla inexpectata*) to *Lonchophylla mordax*.

**Figure 4.**
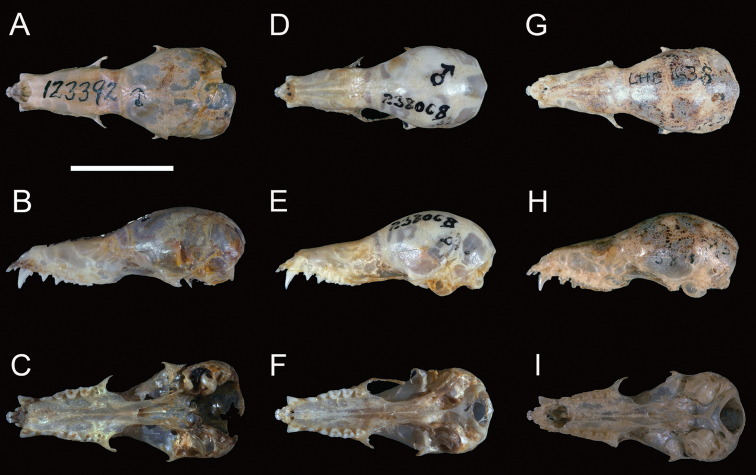
Dorsal (above), lateral (middle), and ventral (below) views of the skull of *Lonchophylla mordax* (**A–C** [USNM 123392, paratype]), *Lonchophylla inexpectata* (**D–F** [USNM 238008, holotype]), and *Lonchophylla dekeyseri* (**G–I** [USNM 584472]). Scale bar: 10 mm.

**Figure 5.**
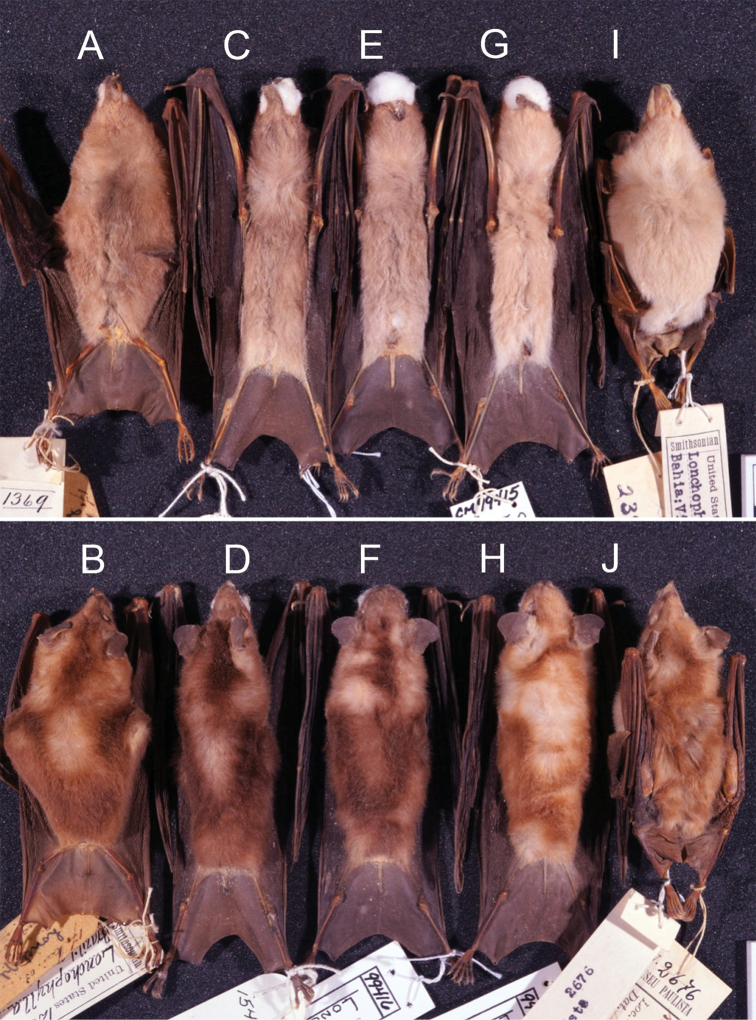
Ventral (above) and dorsal (below) pelage colours of L. mordax **A, B** (USNM 123392, paratype), and *Lonchophylla inexpectata* **C, D** (CM 99432) **E, F** (CM 99416) **G, H** (CM 99415) **I, J** (USNM 238008, holotype).

*Lonchophylla inexpectata* averages significantly smaller than *Lonchophylla mordax* in all cranial dimensions except in POB and BCB (Table 1, Figure 4). This is particularly notable in the length of the mandible (MAL x¯ = 15.6 mm, range [*R*] = 14.1–16.3 mm [*inexpectata*] versus x¯ = 16.1 mm, *R* = 15.5–17.0 mm [*mordax*]). *Lonchophylla inexpectata* can also be distinguished by the ventral pelage, which varies from whitish (e.g., USNM 238008, CM 99415) to pale greyish (near Avelaneous [e.g., CM 99432, 99437]), but near Buffy Brown in *Lonchophylla mordax* (e.g., BM 1903.9.5.34, USNM 123392). The throat and the posterior region of the belly are consistently paler, tending to whitish, in *Lonchophylla inexpectata* (Figure 5).

**Figure 6.**
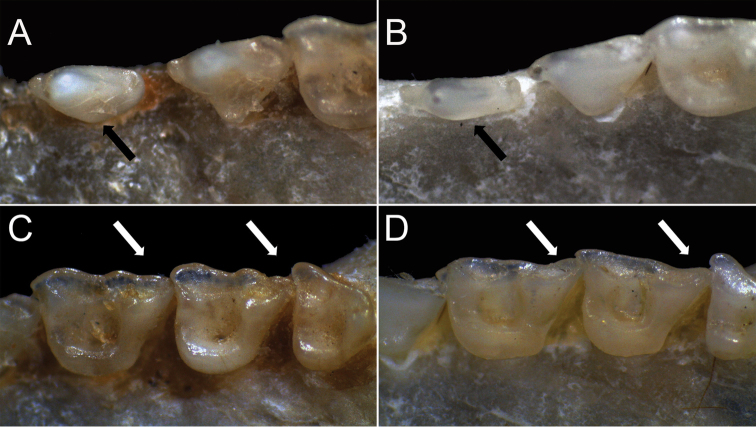
Upper dentition of *Lonchophylla dekeyseri* **A, C** (LDM 3185) and *Lonchophylla mordax* **B, D** (ALP 6149). **A, B** Moderate inner lobe in the first upper premolar (P3) of *Lonchophylla dekeyseri* **A** contrasting with the lingual lobe of P3 absent or very reduced in *Lonchophylla mordax* **B** (similar condition observed in *Lonchophylla inexpectata*) **C, D** metastyles of M1 and M2 reduced or absent in *dekeyseri* **C** contrasting with the metastyles well developed and distinct in *Lonchophylla inexpectata* and *Lonchophylla mordax* **D**.

*Lonchophylla inexpectata* resembles *Lonchophylla dekeyseri* in the pelage colour, but these species can be distinguished by qualitative and quantitative cranial characteristics. *Lonchophylla inexpectata* is significantly larger than *Lonchophylla dekeyseri* in all length measurements of skull and rostrum (GLS, CIL, BAL, MTL, M1M3, MAL, MAN), but *Lonchophylla dekeyseri* averages slightly larger in those measurements of the width of skull and rostrum (BAC, POB, BCB, MAB), indicating a longer but narrower skull in *Lonchophylla inexpectata* (Table 1). *Lonchophylla inexpectata* can be distinguished from *Lonchophylla dekeyseri* by the narrower first upper premolar (P3) in occlusal view, with lingual lobe absent or obsolete (in contrast with the usually more robust P3, which has a small or moderately developed inner lobe in *dekeyseri* [Figure 6]); absence of a deep longitudinal groove in the posterior surface of the canine; narrower and uninflated rostrum, with more widely projecting lacrimals (wider and more inflated rostrum, and lacrimal region almost indistinguishable in *dekeyseri*); upper molars (M1 and M2) with low crowns in lateral view (molars with higher crowns in *dekeyseri*); parastyle of M1 projecting labially over the posterior labial margin of the last upper premolar (P4); mesostyle of M1 shorter; metastyle of M1 well developed (reduced or absent in *dekeyseri* [Figure 6]); parastyle of M2 well developed but slender (well developed and more rounded in *dekeyseri*); mesostyle of M2 shorter; metastyle of M2 distinct, moderate or well developed (reduced or absent in *dekeyseri*).

#### Multivariate analysis.

**Figure 7.**
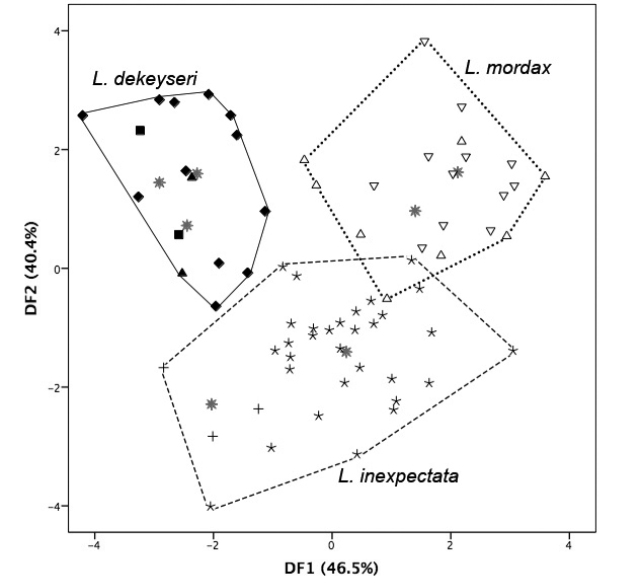
Plots of multivariate individual scores in the first two discriminant functions (DF1, DF2). Samples: *Lonchophylla dekeyseri* (Goiás [black diamonds, *N* = 12]; Mato Grosso do Sul [black squares, *N* = 2]; Distrito Federal [black triangles, *N* = 2]), *Lonchophylla inexpectata* (Barra, Bahia [crosses, *N* = 3]; Exu, Pernambuco [stars, *N* = 31]), and *Lonchophylla mordax* (Itabaiana, Sergipe [white triangles, *N* = 8]; Grota do Angico, Sergipe [white inverted triangles, *N* = 12]). Centroid groups are marked with grey asterisks.

To test the results obtained from the morphological analyses, we performed a discriminant function analysis including samples we confidently assigned to *Lonchophylla dekeyseri* (three groups from the Cerrado of Mid-western Brazil), *Lonchophylla inexpectata* (two groups from the Caatinga of NE Brazil), and *Lonchophylla mordax* (one group from the Caatinga of NE Brazil, and one group from the Atlantic Forest–Caatinga ecotone in NE Brazil). The first two discriminant functions (DF1, DF2) summarized 47% and 40% of the total variation, respectively (Table 2). All samples grouped as expected, confirming the cohesive pattern retrieved from the morphological analysis. Centroids for samples assigned to *Lonchophylla inexpectata* were distinct from those of *Lonchophylla dekeyseri* and *Lonchophylla mordax* across the first two axes, and only a few scores of *Lonchophylla inexpectata* are within the dispersal cloud of *Lonchophylla mordax* (Figure 7). The three species overlap partially across the first axis, but *Lonchophylla inexpectata* distinguishes from *Lonchophylla dekeyseri* and *Lonchophylla mordax* along the second axis. Scores for *Lonchophylla inexpectata* had very low positive to high negative values along the DF2, whereas those for *Lonchophylla dekeyseri* and *Lonchophylla mordax* have low negative to high positive values along this axis.

**Table 2.** Vector correlation coefficients (loadings) between original variables and discriminant functions (DF1, DF2) for samples of *Lonchophylla dekeyseri*, *Lonchophylla inexpectata* and *Lonchophylla mordax*.

	DF1	DF2
Characters	46.5%	40.4%
GLS	0.724	0.021
CIL	0.706	-0.130
MAB	0.240	0.388
BCB	0.268	0.413
POB	-0.149	0.261
BAC	0.117	0.336
BAM	0.413	0.193
M1M3	0.226	0.477
MTL	0.523	0.151
MAL	0.645	0.100

#### Etymology.

The name “*inexpectata*” is Latin for “unexpected”, in allusion to the unexpected (at least for the authors) new taxonomic status of pale-venter populations of *Lonchophylla* from the Caatinga of North-eastern Brazil.

### Key to the Brazil’s species of *Lonchophylla*

**Table:** 

1	Proximal portion of the dorsal surface of the forearm covered with fur; upper canines distinctly grooved along the anterior surface; P4 narrow in occlusal view, with inner lobe reduced and lingual root displaced posteriorly	**2**
–	Proximal portion of the dorsal surface of the forearm not conspicuously furred; upper canines lacking a groove along the anterior surface; P4 robust, with inner lobe well developed and lingual root in the median portion of the tooth	**3**
2	Smaller size; forearm length 37 mm or less; pale-brownish ventral fur; tip of the tragus rounded; parastyles, mesostyles and metastyles of M1 and M2 absent or poorly developed	***Lonchophylla peracchii***
–	Larger size; forearm length 39 mm or more; pale-greyish ventral fur; tip of the tragus pointed; parastyles, mesostyles and metastyles of M1 and M2 well developed	***Lonchophylla bokermanni***
3	P3 robust in occlusal view, with lingual lobe varying from small to moderately developed projection; presence of a conspicuous longitudinal groove along the posterior surface of the canine; metastyle of M1 and M2 absent or reduced	***Lonchophylla dekeyseri***
–	P3 narrow in occlusal view, usually without inner lobe or with a reduced lobe; absence of a conspicuous longitudinal groove along the posterior surface of the canine; metastyle of M1 and M2 distinct and developed	**4**
4	Ventral fur pale-brownish; mandibular length 15.5–17.0 mm	***Lonchophylla mordax***
–	Ventral fur whitish or pale-greyish on the throat and abdomen (particularly on the posterior region of the belly); mandibular length 14.1–16.3 mm	***Lonchophylla inexpectata***

## Discussion

**Historical remarks.** Previous assignments of *Lonchophylla inexpectata* to *Lonchophylla mordax* seem to have originated with Lima (1926: 36) who based his account of *Lonchophylla mordax* on the series from Barra, which was collected by Garbe and deposited in the Museu de Zoologia da Universidade de São Paulo. Barra is in the *sertão* of Bahia (Caatinga), ca. 450–500 km west of Lamarão, which is in the *agreste* of Bahia (transition between Atlantic Forest and Caatinga; type locality of *Lonchophylla mordax*). Thomas (1903: 459) described *Lonchophylla mordax* as follows:

*General external appearance, so far as can be judged by skins, exactly as in* Glossophaga soricina, *except that the colour averages paler. The type is near “cinnamon-brown” above, the bases of the hairs whitish, and “wood-brown” below, but there is some variation in tone, and the darker specimens are quite as dark as the paler examples of* Glossophaga *obtained at the same place*.

Lima (1926) seems to have misinterpreted Thomas (1903) where he reported that “darker specimens [of *Lonchophylla mordax*] are quite as dark as the paler examples of *Glossophaga* obtained at the same place.” Lima’s conclusion might be biased by the series he had at hand, primarily composed by pale-venter specimens from Barra, Bahia. However, at that time, *Lonchophylla mordax* was unquestionably the closest species—geographically and morphologically. Although Lima had identified this series from Barra as *Lonchophylla mordax*, the label of the USNM 238008 bears the notation “*Subsp. n.*?”

Vieira (1942: 321) followed Lima (1926) and based his account of *Lonchophylla mordax* on the same specimens collected by Garbe. Both recognized *Lonchophylla mordax* as a pale-venter species. This was followed by Taddei et al. (1983) who compared the species they were describing (*Lonchophylla dekeyseri*) with “*Lonchophylla mordax*”—the other pale-venter species from NE Brazil, according to those authors. However, according to Thomas (1903), the ventral pelage of *Lonchophylla mordax* is “wood-brown”, but with some variation, with darker specimens almost as dark as paler specimens of *Glossophaga* from the same area. *Glossophaga soricina* (Phyllostomidae)—the only species of the genus that occur in the region—has ventral pelage varying from “buffy to fuscous” (Alvarez et al. 1991).

**Taxonomic remarks.** Molecular and morphological analyses have recovered *Lonchophylla* (sensu Griffiths and Gardner 2008) as a paraphyletic assemblage (Dávalos and Jansa 2004, Woodman and Timm 2006, Woodman 2007). Combining evidence from nuclear and mitochondrial genes, karyotypes and skull morphology, Parlos et al. (2014) also retrieved *Lonchophylla* as paraphyletic. Based on their findings, Parlos et al. (2014) described *Hsunycteris* and moved three species into this new genus—*thomasi* J. A. Allen, 1904; *cadenai* Woodman & Timm, 2006; and *pattoni* Woodman & Timm, 2006. As a result, *Lonchophylla* comprised 12 South and Central American species (Parlos et al. 2014). However, several species were not assessed, including *Lonchophylla mordax*—the type species of *Lonchophylla*. According to Parlos et al. (2014), the two genera can be distinguished by size (with species in *Lonchophylla* larger than those in *Hsunycteris*), qualitative cranial features, and karyotypes (*Lonchophylla* spp.: diploid number [2n] = 48, fundamental autosomal number [NF] = 50; *Hsunycteris* spp.: 2n = 30–36, NF = 34–50).

The samples we have available show that *Lonchophylla dekeyseri* and *Lonchophylla mordax* are in parapatry with *Lonchophylla inexpectata*: *Lonchophylla dekeyseri* occurs in the Cerrado of Brazil and possibly in the Bolivian savannah (USNM 584472, 584473) and the Cerrado–Caatinga ecotone in NE Brazil (DZSJRP 11459); and *Lonchophylla mordax* occurs in the Atlantic Forest–Caatinga ecotone (*agreste*), and along the eastern border of the Caatinga (*sertão*). We are not convinced that *Lonchophylla dekeyseri* occurs in the Bolivian savannah and in the Cerrado–Caatinga ecotone in NE Brazil. One of the specimens supporting these records was examined a long time ago (DZSJRP 11459), and the other two (USNM 584472, 584473) are distinct from other samples of *Lonchophylla dekeyseri* as determined in a previous discriminant function analysis. These specimens are not included in this analysis because we were not able to compare them with samples from other localities. Records previously assigned to *Lonchophylla mordax* from N Brazil are based primarily on Handley (1967) and Piccinini (1974), and those identifications were not confirmed in subsequent surveys. We speculate that they are misidentifications of *Lonchophylla thomasi*, now *Hsunycteris thomasi*. Similarly, previous unvouchered records of *Lonchophylla mordax* from the Atlantic Forest of SE Brazil apparently represent *Lonchophylla peracchii* based on the identity of material we have examined from nearby localities.

After Parlos et al.’s (2014) assignment of *Lonchophylla thomasi* J. A. Allen, 1904 to *Hsunycteris*, *Lonchophylla inexpectata* is the fifth *Lonchophylla* reported from Brazil—all pending phylogenetic positioning. There are several specimens pending verification of identity, particularly those from the Caatinga. Additional material, particularly from NE and Mid-western Brazil, will be important to a clearer understanding of the taxonomic diversity, and the geographic distribution of Brazilian species of *Lonchophylla*.

## Supplementary Material

XML Treatment for Lonchophylla inexpectata

## Appendix

Specimens examined. Abbreviations for collections are in “Methods”.

*Lonchophylla bokermanni* (08): Brazil, Minas Gerais: Diamantina (18°23'S, 43°61'W: MN 79996, MN 79997); Serra do Cipó (19°16'S, 43°36'W: DZSJRP 10342 [paratype], 10347 [holotype], 10408 [paratype], 11410 [paratype], 11411 [paratype], 10412 [paratype; referred in the original description as ZUEC 585]).

*Lonchophylla dekeyseri* (16): Brazil, Distrito Federal: Parque Nacional de Brasília (15°41'S, 47°59'W: DZSJRP 10099 [holotype]); unknown locality (ALP 6706, 6707). Brazil, Goiás: Mambaí (14°29'S, 46°06'W: LDM 283, 3008, 3065, 3066, 3104, 3169, 3170, 3184, 3185, 3201, 3215, 3270). Brazil, Mato Grosso do Sul: Corumbá (19°61'S, 57°45'W: LDM 2642).

Lonchophylla cf. dekeyseri (3): Bolivia, Santa Cruz: Velasco (13°54'27"S, 60°48'52.92"W: USNM 584472, 584473). Brazil, Piauí: Sete Cidades, Piracuruca (03°56'S, 41°44'W: DZSJRP 11459 [paratype of *dekeyseri*]).

*Lonchophylla inexpectata*: Brazil, Bahia (43): Barra (12°42'S, 41°33'W: USNM 238008 [holotype], AMNH 235608, FMNH 21077, 21078 [paratypes]). Brazil, Pernambuco: Buíque, Serra do Catimbau (08°37'S, 37°09'W: FMNH 137414 [paratype]); 17 km south of Exu (07°41'S, 39°32'W: CM 99413–99450).

Lonchophylla cf. inexpectata (1): Brazil, Bahia: Andaraí, unknown locality (ALP 3686).

*Lonchophylla mordax* (35): Brazil, Bahia: Lamarão (11°45'S, 38°55'W: BM 1903.9.5.34 [holotype], USNM 123392 [paratype]). Brazil, Sergipe: Itabaiana (10°68'S, 37°42'W: ALP 6149, 8769, 8770, 8812–8819); Parque Nacional Grota do Angico (09°65'S, 37°67'W: ALP 9747, 9752, 9755, 9757, 9759, 9761, 9762, 9768, 9769, 10075–10082, 10084–10088).

*Lonchophylla peracchii* (75): Brazil, Espírito Santo: Sooretama, BR-101, Km 105, Reserva Biológica de Sooretama (19°1'48.97"S, 40°1'8.976” W: UFES 2046, 2047, 2117) Brazil, Rio de Janeiro: Angra dos Reis, Ilha da Gipóia (23°02'S, 44°21'W: LDM 4200, 4423); Angra dos Reis, Ilha Grande (23°10'S, 44°12'W: DZSJRP 15159 [paratype], 15160, 15161, 15162 [holotype], 15163 [paratype], LDM 246, 2090, 3450, 3896, 3897, 4052, 4233, 4533); Cambuci (21°34'S, 41°54'W: LDM 4250, 4253, 4477); Casimiro de Abreu, Morro de São João (22°29'S, 41°58'W: LDM 2219, 2245, 4113, 4222, 4226, 4227); Itaguaí, Ilha de Itacuruçá (23°56'S, 43°53'W: LDM 5085); Mangaratiba, Vale do Rio Sahy (23°55'S, 43°59'W: LDM 5128); Nova Iguaçú, Reserva Biológica do Tinguá (22°39'S, 43°34'W: ALP 6265, 6560, 6561, 6283, 6284, 6556, 6656–6559); Parati (23°19'S, 44°38'W: LDM 996, 997); Rio de Janeiro, Estrada Rio-Santos (23°55'S, 43°16'W: LDM 5008, 5010); Rio de Janeiro, Floresta da Tijuca (22°57'S, 43°24'W: LDM 1064, 1460); Rio de Janeiro, Jardim Botânico (22°58'S, 43°13'W: LDM 875); Rio de Janeiro, Parque Estadual da Pedra Branca (22°52'S, 43°23'W: ALP 5664, 5820, 5860); Rio de Janeiro, Reserva do Grajaú (22°55'S, 43°16'W: ALP 1783–1785, LDM 237, 238, 246–248, 250, 270, 280, 281, 345, 395, 531–533, 1359, 1495–1497, 1499); Rio de Janeiro, Reserva Rio das Pedras (22°59'S, 44°06'W: LDM 1781, 3700); Teresópolis, Parque Nacional da Serra dos Órgãos (22°26'S, 42°59'W: ALP 6482). Brazil, São Paulo: Ubatuba, Picinguaba (23°18'S, 44°53'W: ALP 10242).

## Supplementary materials

Supplementary material 1 Occurrence localities for Bolivian and Brazilian species of *Lonchophylla* Data type: Occurrence localities This dataset is made available under the Open Database License (http://opendatacommons.org/licenses/odbl/1.0/). The Open Database License (ODbL) is a license agreement intended to allow users to freely share, modify, and use this Dataset while maintaining this same freedom for others, provided that the original source and author(s) are credited.Ricardo Moratelli, Daniela Dias
